# Drinking hydrogen water improves photoreceptor structure and function in retinal degeneration 6 mice

**DOI:** 10.1038/s41598-022-17903-8

**Published:** 2022-08-10

**Authors:** Tsutomu Igarashi, Ikuroh Ohsawa, Maika Kobayashi, Kai Miyazaki, Toru Igarashi, Shuhei Kameya, Asaka Lee Shiozawa, Yasuhiro Ikeda, Yoshitaka Miyagawa, Mashito Sakai, Takashi Okada, Iwao Sakane, Hiroshi Takahashi

**Affiliations:** 1 Department of Ophthalmology, Nippon Medical School Chiba Hokusoh Hospital, 1715, Kamakari, Inzai, Chiba 270-1694 Japan; 2 Department of Ophthalmology, Nippon Medical School, 1-1-5 Sendagi, Bunkyo-ku, Tokyo, 113-8602 Japan; 3 Department of Biochemistry and Molecular Biology, Nippon Medical School, 1-1-5 Sendagi, Bunkyo-ku, Tokyo, 113-8602 Japan; 4 Biological Process of Aging, Tokyo Metropolitan Institute of Gerontology, 35-2 Sakae-cho, Itabashi-ku, Tokyo, 173-0015 Japan; 5 Faculty of Medicine, Nippon Medical School, 1-1-5 Sendagi, Bunkyo-ku, Tokyo, 113-8602 Japan; 6 Department of Pediatrics, Nippon Medical School, 1-1-5 Sendagi, Bunkyo-ku, Tokyo, 113-8602 Japan; 7 Faculty of Medicine, University of Miyazaki, 5200 Kihara, Kiyotake, Miyazaki, Miyazaki 889-1692 Japan; 8 Division of Molecular and Medical Genetics, Center for Gene and Cell Therapy, Institute of Medical Science, University of Tokyo, 4-6-1, Shirokanedai, Minato-ku, Tokyo, 108-8639 Japan

**Keywords:** Experimental models of disease, Hereditary eye disease

## Abstract

Retinitis pigmentosa (RP) is a genetically heterogeneous group of inherited retinal disorders involving the progressive dysfunction of photoreceptors and the retinal pigment epithelium, for which there is currently no treatment. The rd6 mouse is a natural model of autosomal recessive retinal degeneration. Given the known contributions of oxidative stress caused by reactive oxygen species (ROS) and selective inhibition of potent ROS peroxynitrite and OH·by H_2_ gas we have previously demonstrated, we hypothesized that ingestion of H_2_ water may delay the progression of photoreceptor death in rd6 mice. H_2_ mice showed significantly higher retinal thickness as compared to controls on optical coherence tomography. Histopathological and morphometric analyses revealed higher thickness of the outer nuclear layer for H_2_ mice than controls, as well as higher counts of opsin red/green-positive cells. RNA sequencing (RNA-seq) analysis of differentially expressed genes in the H_2_ group versus control group revealed 1996 genes with significantly different expressions. Gene and pathway ontology analysis showed substantial upregulation of genes responsible for phototransduction in H_2_ mice. Our results show that drinking water high in H_2_ (1.2–1.6 ppm) had neuroprotective effects and inhibited photoreceptor death in mice, and suggest the potential of H_2_ for the treatment of RP.

## Introduction

Retinitis pigmentosa (RP) is a genetically heterogeneous group of inherited retinal disorders characterized by diffuse progressive dysfunction of predominantly rod photoreceptors, with subsequent degeneration of cone photoreceptors and the retinal pigment epithelium (RPE)^1^. Visual impairment usually manifests as night blindness and progressive visual field loss. The prevalence of RP is 1:3000–1:5000^2,3^, and approximately 1.4 million people worldwide are affected by RP. Currently, no treatment options are available for patients with RP, and disease progression to blindness is unavoidable. Over 100 different gene mutations have been identified as factors that influence the development and progression of RP^4^. Although genetic mutations trigger RP, disease progression is affected by microenvironmental changes associated with retinal degeneration such as oxidative stress^5,6^, and inflammation^7,8^.

Oxidative stress causes various neurological disorders of the retina^9^. Oxidative damage leads to cone cell death, and antioxidants reduce oxidative damage and promote cone survival and function^10^. Compared to people without RP, those with RP have increased carbonyl content and a decreased ratio of reduced to oxidized glutathione^10^. Oxidative stress is derived from reactive oxygen species (ROS), such as hydroxyl radicals (OH·), superoxide anion radicals (O_2_^−·^), hydrogen peroxide (H_2_O_2_), and nitric oxide. We reported that molecular hydrogen (H_2_) selectively reduces the extremely toxic ROS OH· and peroxynitrite, but not O_2_^−·^, H_2_O_2_, or nitric oxide^11^. Moreover, administration of H_2_ gas markedly suppresses brain ischemia–reperfusion injury^11^ and retinal ischemia–reperfusion injury^12^. ROS is suspected to be quite important in neurodegenerative diseases such as Parkinson’s disease (PD), Alzheimer’s disease (AD), and Huntington’s disease. ROS-induced mitochondrial damage is associated with the triggers of PD, AD, and other neurodegenerative diseases^13^. H_2_-dissolved water (H_2_ water) reduces dopaminergic neuronal cell loss and downregulates 4-hydroxy-2-nonenal, which is an oxidative stress marker, in dopaminergic neurons in PD animal models, compared with normal water^14,15^. These results suggested that the intake of H_2_ water reduces neurotoxic damage even after chronic toxin administration. Previous studies have demonstrated that H_2_ water is safe to drink. No significant differences were seen between control and hydrogen groups in terms of food or water consumption, body weight, or growth pattern during 12 months in rats^16^. Moreover, H_2_ water was well tolerated and caused no adverse effects during 48 weeks of administration in humans^7^.

Neuroinflammation is widely associated with and contributes to various forms of neurodegeneration, including RP^6,17,18^. Microglia in human RP patients become reactive in response to signals from degenerating rods and migrate to the photoreceptor layers^19^. RP model mice display widespread microglial activation^20^ and reactive gliosis is featured by the increased expression of glial fibrillary acidic protein (GFAP) in macroglial cells^21^. Microglial cell activation was observed prior to the initiation of photoreceptor death and inhibition of microglial cells improved photoreceptor survival and morphology^22^. H_2_ was recently reported to inhibit microglial activation in acute neuroinflammation models such as the mouse middle cerebral artery occlusion model^23^ and the rat traumatic brain injury model^24^.

*MFRP* is expressed in the RPE and ciliary bodies^25^ and mutations of *MFRP* causes microphthalmia, high hyperopia, foveoschisis, areas of RPE atrophy, and optic disc drusen in humans^26–29^. MFRP-deficient eyes have spots of retinal discoloration and reduced electroretinogram (ERG) readings.

Well-characterized animal models exist, and understanding of the genetic basis of the disease is increasing^30^. The rd6 mouse is a natural model of autosomal recessive retinal degeneration, and is caused by a 4-bp deletion in a splice donor site in *Mfrp*^25,31^. In rd6 mice, slowly progressive retinal degeneration affects both rod and cone cells beginning from 3–4 weeks of age, soon after the retina develops. Slow, progressive loss of the photoreceptors occurs over approximately 16 months. Although MFRP protein function is not completely understood^32,33^, MFRP is known to regulate the lipidome and transcription for photoreceptor function^34^. Furthermore, retinal degeneration is reportedly caused by *MFRP* mutations in humans^35^.

In this study, we investigated whether H_2_ water could reduce and delay the progression of photoreceptor death in rd6 mice.

## Results

### Preservation of the H_2_ concentration in H_2_ water

**Figure 1:**
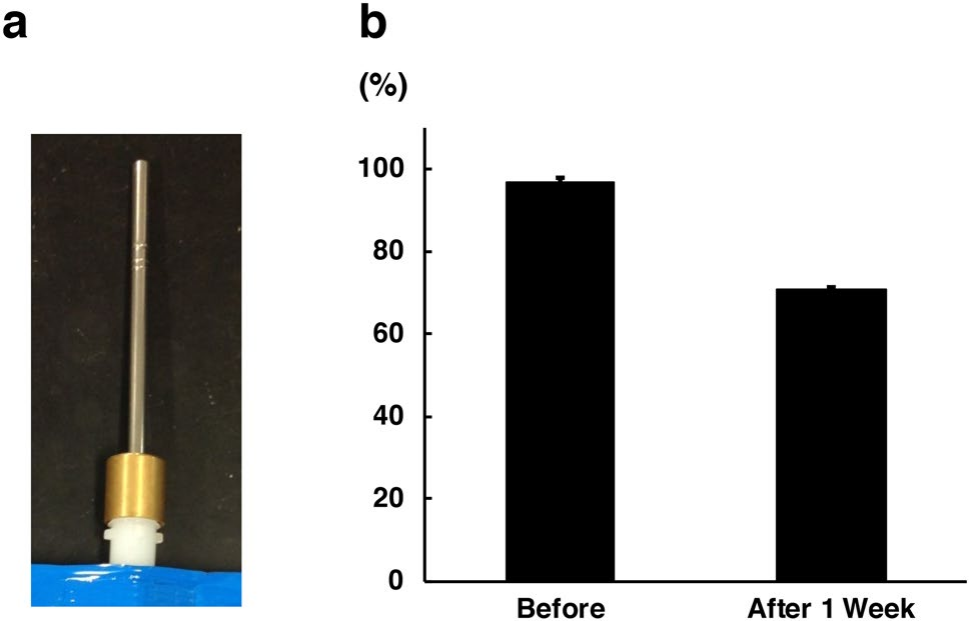
Preservation of H_2_ concentration in H_2_ water. (**a**) To maintain a high concentration of H_2_ in water, we developed water drinking valves. (**b**) The hydrogen concentration was 96.84 ± 1.03% before drinking and 70.68 ± 0.31% 1 week after drinking (C57BL/6J mice, n = 4). Bars depict mean ± standard deviation (SD).

To ensure that mice drank water with a stable, high concentration of H_2_, we developed unique water drinking valves designed to preserve H_2_ (Fig. 1a). The average amount of water consumed per mouse was 3.42 ± 0.14 ml/day. The hydrogen concentration before drinking was 96.84 ± 1.03%, and was maintained at 70.68 ± 0.31% in the first week after drinking (C57BL/6J mice, n = 4) (Fig. 1b).

### Hydrogen prevents outer retina thinning in rd6 mice

**Figure 2:**
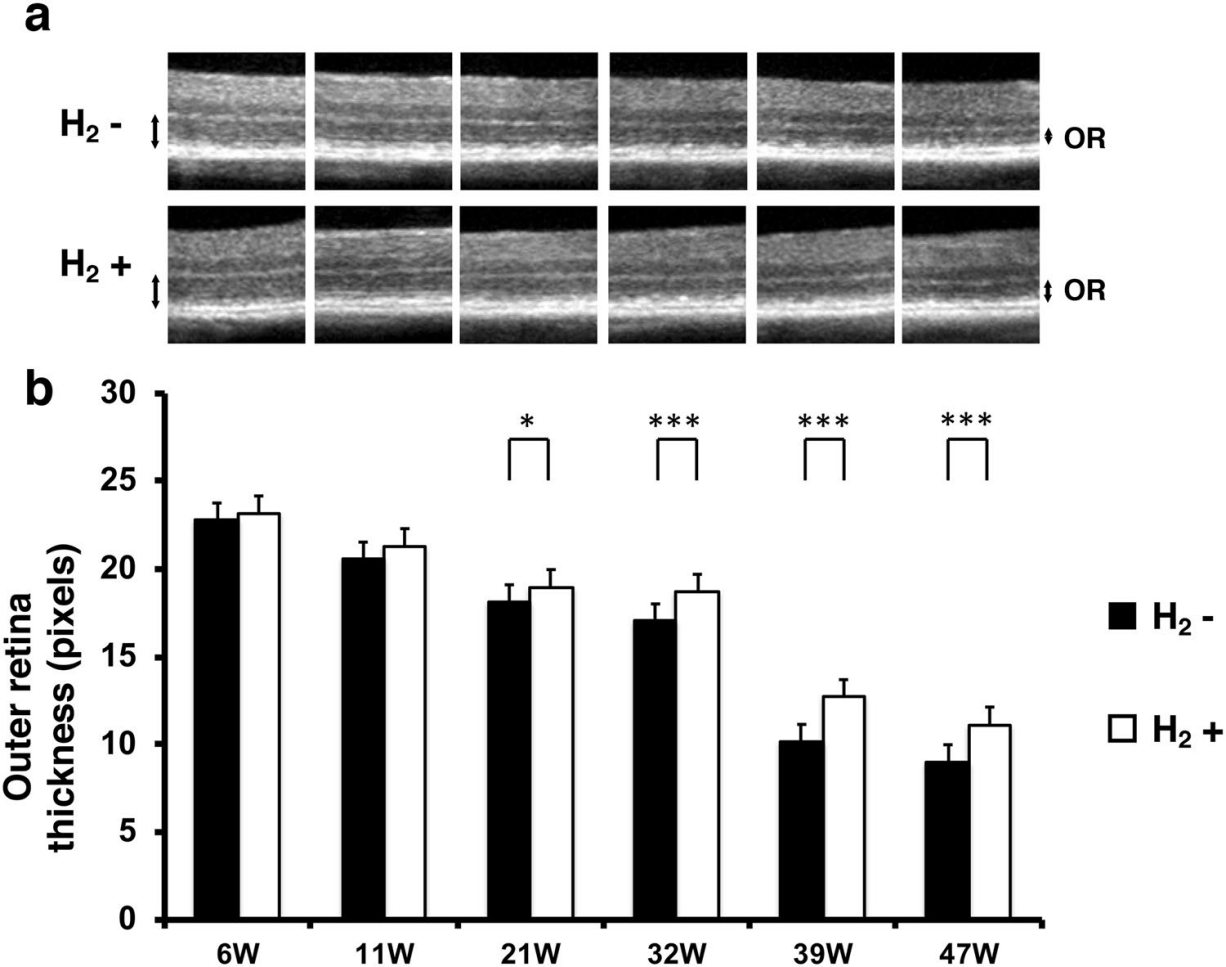
Effect of H_2_ water on outer retinal thickness. (**a**) Representative retinal projections of OCT scans at 6–47 weeks of age. Double-headed arrows show the outer retina. *OR* outer retina. (**b**) Quantification of the outer retina thickness with/without H_2_ water. The outer retina thickness with H_2_ water (n = 10) was significantly greater than that without H_2_ water (n = 8) (p < 0.001 and 0.05). Bars depict mean ± standard deviation (SD). *p < 0.05, ***p < 0.001.

In rd6 mice, photoreceptor degeneration occurs from 3–4 weeks of age, and progressive loss of the photoreceptor outer segments continues over approximately 16 months^31^. To examine whether H_2_ water prevents thinning of the outer retina in rd6 mice, we measured outer retinal thickness using optical coherence tomography (OCT), a non-invasive imaging modality that produces cross-sectional reflectance images of the retina. Figure 2a shows representative images for the control and H_2_ groups. Outer retinal thicknesses (in pixels) of the control group (n = 8) and H_2_ group (n = 10), respectively, at different postnatal ages were as follows: 6 weeks (22.78 ± 1.88 vs 23.16 ± 2.04, p = 0.35), 11 weeks (20.55 ± 1.56 vs 21.32 ± 0.85, p = 0.1), 21 weeks (18.11 ± 0.92 vs 18.98 ± 0.53, p = 0.012), 32 weeks (17.02 ± 1.03 vs 18.7 ± 0.82, p = 0.00067), 39 weeks (10.15 ± 0.81 vs 12.7 ± 1.45, p = 0.00021), and 47 weeks (8.99 ± 0.58 vs 11.14 ± 1.05, p = 0.00005) (Fig. 2b). OCT imaging showed that the outer retina was significantly thicker in the H_2_ group than in the control group from 21 to 47 weeks postnatally. These results suggest that drinking H_2_ water can prevent outer retinal thinning.

### Hydrogen protects rod function in rd6 mice

**Figure 3:**
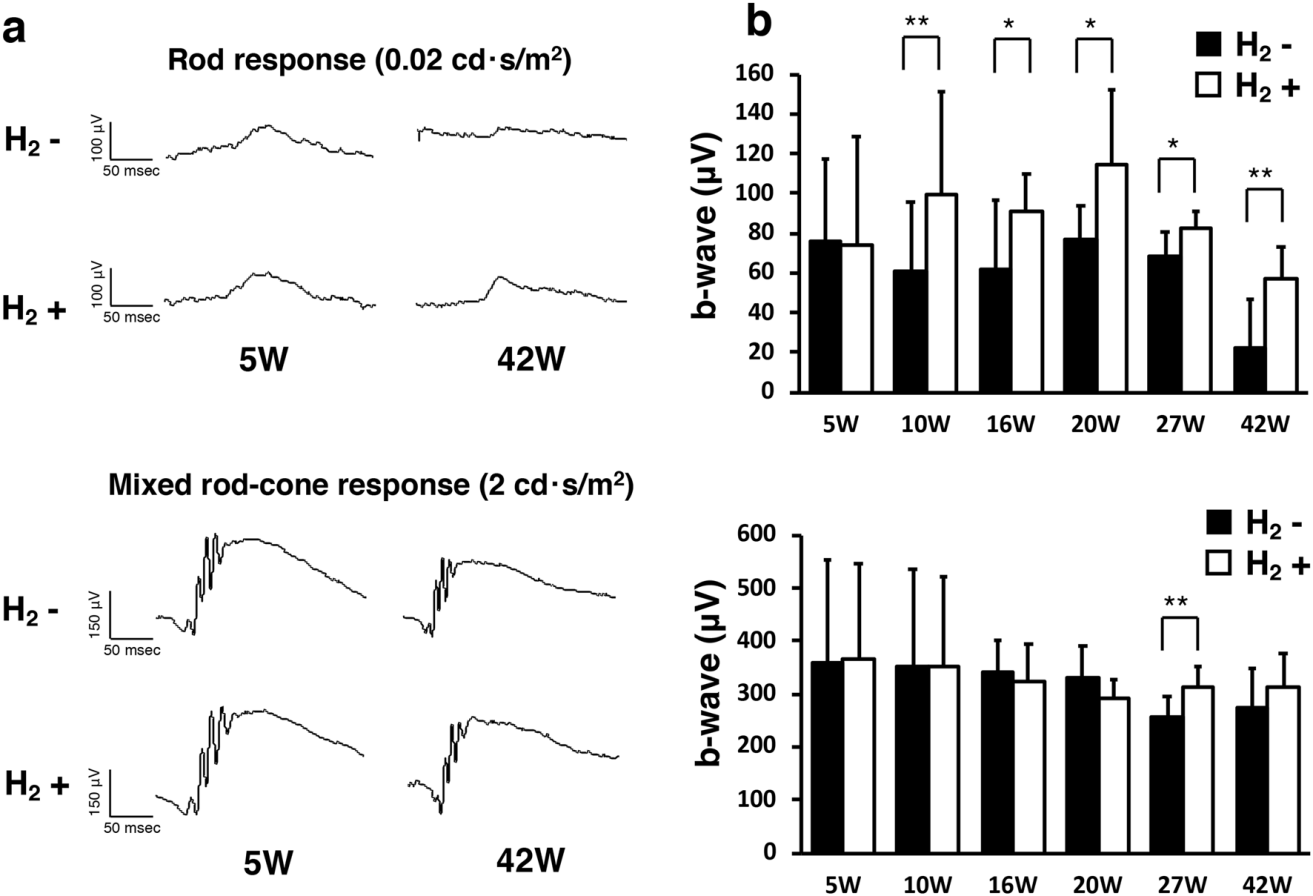
B-wave of ERGs in rd6 mice with/without H_2_ water. (**a**) Representative b-wave with/without H_2_ water at 0.02 cd·s/m^2^ and 2 cd·s/m^2^. (**b**) Quantification of the b-wave amplitude from 5 to 42 weeks of age. We found a significant difference between the control group (n = 8) and H_2_ group (n = 10). Bars depict means ± standard deviation (SD). *p < 0.05, **p < 0.01.

To determine whether preservation of the outer retina results in improved retinal function in the H_2_ group, we performed scotopic ERG to measure rod response and mixed rod-cone response elicited with 0.02 cd·s/m^2^ and 2 cd·s/m^2^ stimuli, respectively (H_2_ group: n = 10; control group: n = 8). Figure 3a shows representative ERG recordings of the b-wave amplitudes at 0.02 cd·s/m^2^ and 2 cd·s/m^2^. The rod response represented by b-waves at 0.02 cd·s/m^2^ was significantly preserved in the H_2_ group beginning at 10 weeks postnatal through 42 weeks (5 W; p = 0.93, 10 W; p = 0.005, 16 W; p = 0.036, 20 W; p = 0.02, 27 W; p = 0.012, 42 W; p = 0.003; Fig. 3b). On the other hand, the mixed rod-cone response reduction in rd6 mice was relatively moderate compared to the rod response, and a significantly higher amplitude of the mixed rod-cone response was observed only at 27 weeks old in the H_2_ group (p = 0.006, Fig. 3b). These results suggest that drinking H_2_ water can rescue rod function in rd6 mice.

### Effect of hydrogen on histopathological changes, cell density in the outer nuclear layer, opsin red/green positivity, and opsin blue positivity in retinal cross sections

**Figure 4:**
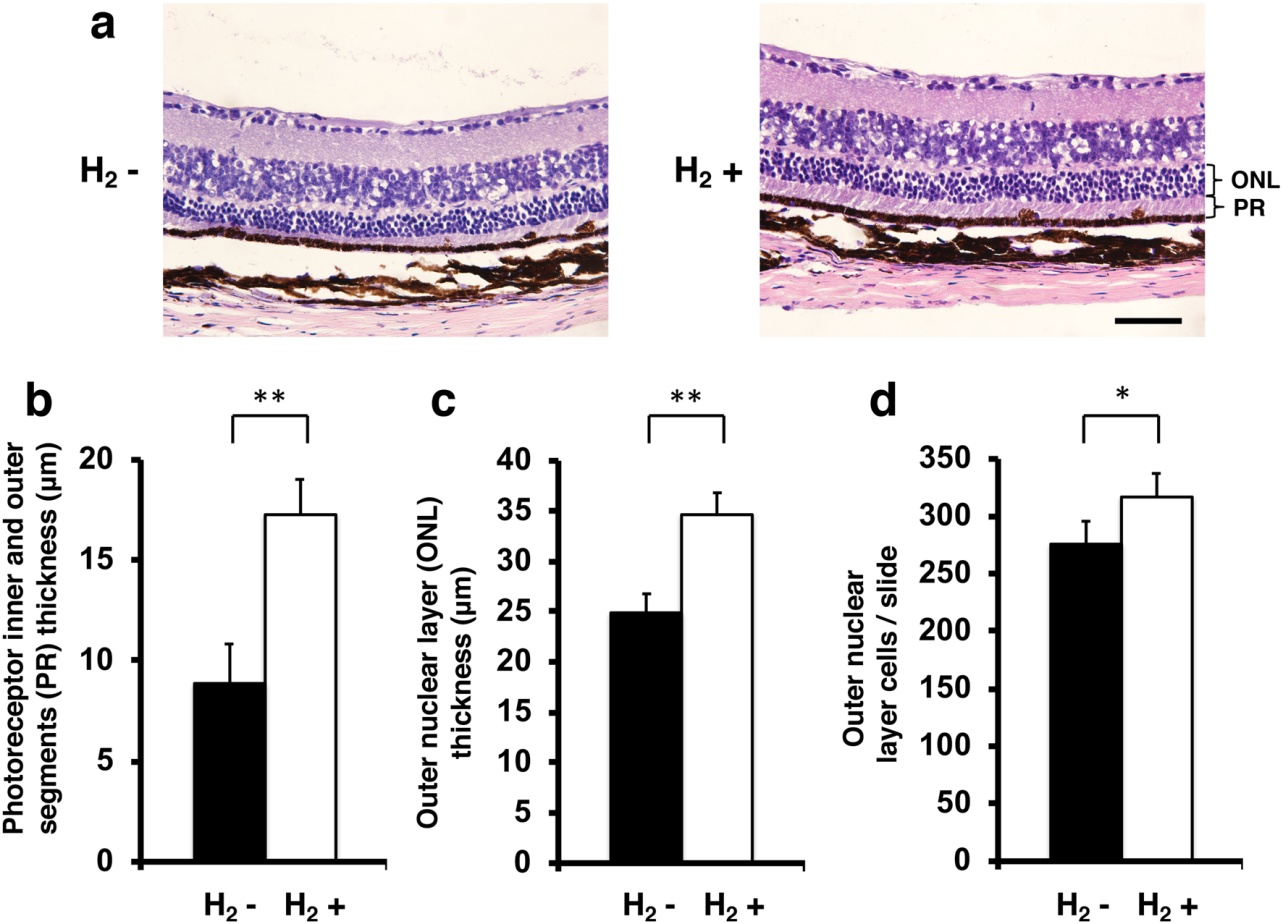
Thickness of photoreceptor inner and outer segments and outer nuclear layer and number of outer nuclear layer cells. (**a**) Images of representative slices from the control group (n = 4) and H_2_ group (n = 4). Photoreceptor inner and outer segments (PR) thickness and outer nuclear layer (ONL) are shown. (**b**) Photoreceptor inner and outer segments thickness with/without H_2_ water. Thickness was significantly greater with H_2_ water than without H_2_ water (p < 0.01). (**c**) Outer nuclear layer (ONL) thickness with/without H_2_ water. Thickness was significantly greater with H_2_ water than without H_2_ water (p < 0.01). (**d**) Outer nuclear layer cells/slide with/without H_2_ water. Thickness was significantly greater with H_2_ water than without H_2_ water (p < 0.05). Bars depict mean ± standard deviation (SD). Scale bar 50 μm. *p < 0.05, **p < 0.01.

To evaluate the protective effect of hydrogen, we then examined histopathological and morphometric changes in 49-week-old rd6 mice. Figure 4a shows images of representative slices from the control group (n = 4) and H_2_ group (n = 4). The photoreceptor inner and outer segments thickness of the control group was 8.8 ± 2 μm, and that of the H_2_ group was 17.2 ± 1.8 μm (p = 0.00039; Fig. 4b). The outer nuclear layer thickness was 24.9 ± 1.9 μm in the control group, and 34.6 ± 2.2 μm in the H_2_ group (p = 0.00027, Fig. 4c). The number of cells per slide in the outer nuclear layer was 275.8 ± 20 in the control group, and 316.8 ± 20.6 in the H_2_ group (p = 0.015; Fig. 4d).

**Figure 5:**
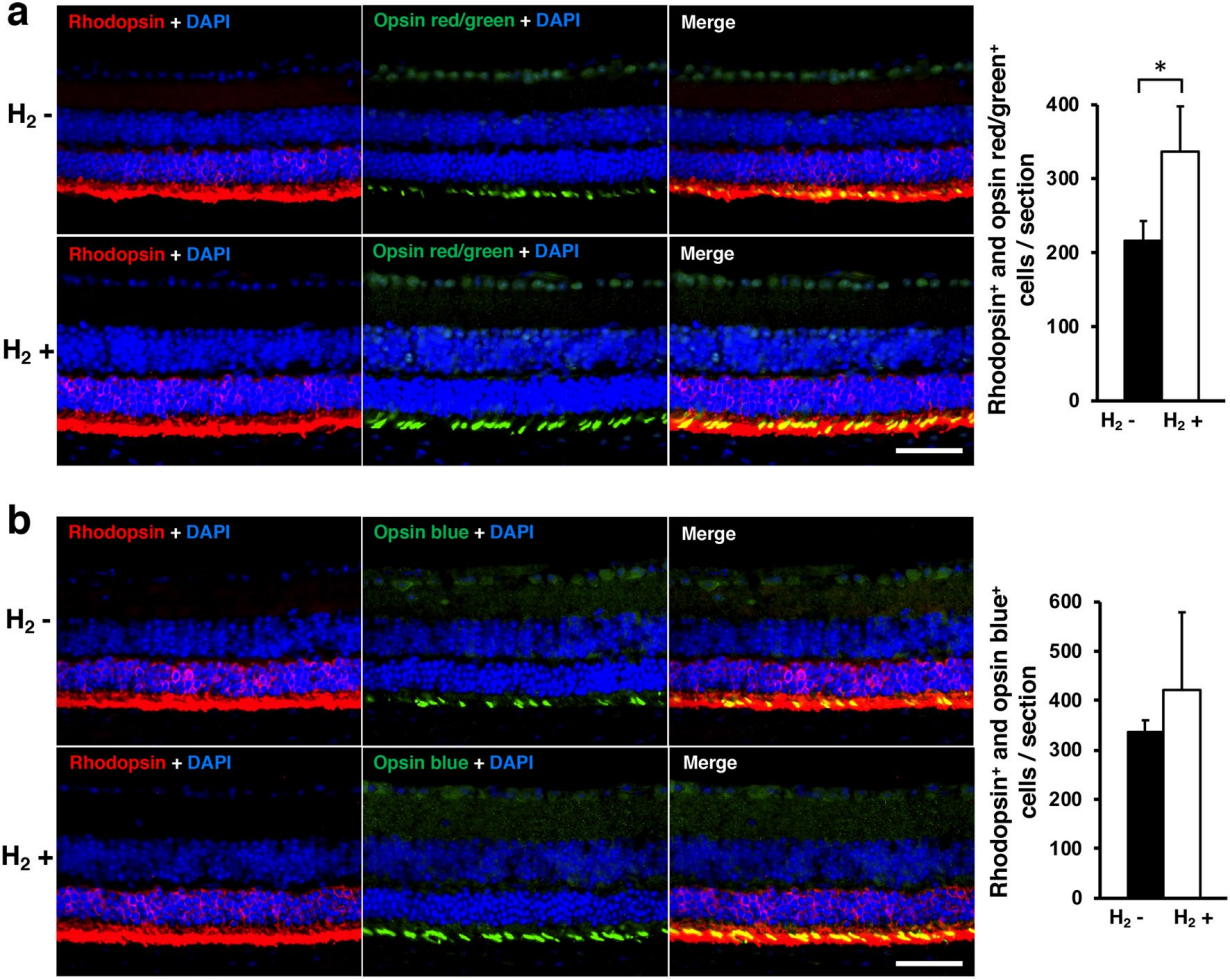
Immunohistochemical analysis of rhodopsin and opsin. (**a**) Immunohistofluorescence co-staining for rhodopsin (red) and opsin red/green (green) is shown in representative retinal specimens with/without H_2_ water. Double-positive cells (yellow) were counted for eye cups in one slide. The number of double-positive cells was significantly greater in the control group (n = 4) than in the H_2_ group (n = 4; p < 0.05). (**b**) Immunohistofluorescence co-staining (yellow) for rhodopsin (red) and opsin blue (green) is shown in representative retinal specimens with/without H_2_ water. No significant difference was found (p = 0.35). Scale bar 50 μm. Bars depict mean ± standard deviation (SD).

The photographs in Fig. 5a,b show representative retinal cross sections with cells stained red for rhodopsin and green for opsin red/green or opsin blue. Nuclei were stained with DAPI (blue). The number of cells positive for both rhodopsin and opsin red/green (yellow in the merged image) per vertical section through the retina was 216.7 ± 26.4 in the control group, and 337.3 ± 60.6 in the H_2_ group (p = 0.037; Fig. 5a). The number of cells positive for both rhodopsin and opsin blue (yellow in the merged image) per vertical section through the retina was 338 ± 22.5 in the control group, and 420.3 ± 158.9 in the H_2_ group (p = 0.35; Fig. 5b). These results suggest that drinking H_2_ water can protect photoreceptor cells and opsin red/green-positive cells in rd6 mice.

### Hydrogen induces high expression of genes involved in phototransduction

**Figure 6:**
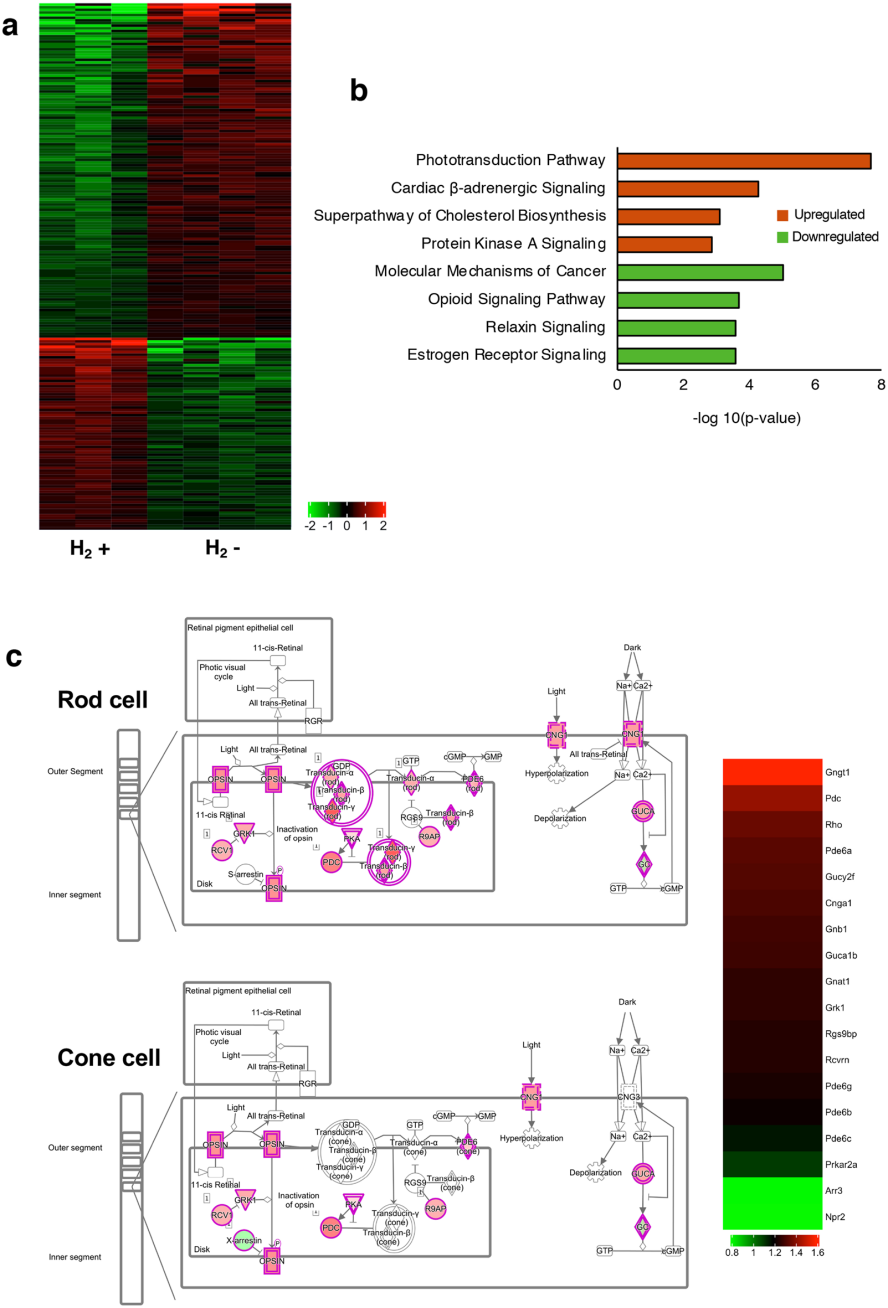
RNA-seq analysis. (**a**) Heatmap based on differentially expressed genes. Each column represents a sample (H_2_ group: n = 3; control group: n = 4), and each row represents a gene. The expression level of each gene in a single sample is depicted according to the color scale. (**b**) Pathway analysis of the differentially expressed genes based on IPA. The top four most significant up- and downregulated pathways after drinking H_2_ water. (**c**) IPA pathway and heatmap of phototransduction. Red and green colors indicate that the genes are upregulated or downregulated, respectively.

To characterize the effects of H_2_ water on gene expression in rd6 mice, we performed whole transcriptome analysis of the neural retina with/without H_2_ water. RNA-seq analysis of differentially expressed genes (DEG) in the H_2_ group (n = 3) vs control group (n = 4) revealed 1996 genes with significantly different expression (upregulation of 856, downregulation of 1140 genes), as indicated in the heatmap (Fig. 6a). To identify signature trends for upregulation or downregulation of downstream pathways, we performed gene and pathway ontology analysis and showed the four most upregulated pathways and the four most downregulated pathways (Fig. 6b). Top molecular pathways differentially regulated following hydrogen drinking included transcriptional changes in approximately 18 genes involved in phototransduction pathways (Fig. 6c). The diagram produced by Ingenuity Pathway Analysis (IPA) illustrates phototransduction in rod cells and cone cells. As illustrated, the majority of genes included in phototransduction were upregulated in the H_2_ group. We also examined the superpathway of cholesterol biosynthesis, another upregulated pathway in the H_2_ group. Genes in this superpathway including *Idl1*, *Acta2*, *Cyp51A1*, and *Hmgcs1* were slightly elevated (see Supplementary Fig. S1 online). Gene ontology groups such as inflammatory response (GO:0006954) and response to oxidative stress (GO:0006979), which were expected to be different, were not significantly changed as a system (Supplementary Fig. S1).

### GFAP, monocytes/macrophages-2 (MOMA-2) positivity in retinal cross-sections and ionized calcium-binding adapter molecule 1 (Iba-1)

**Figure 7:**
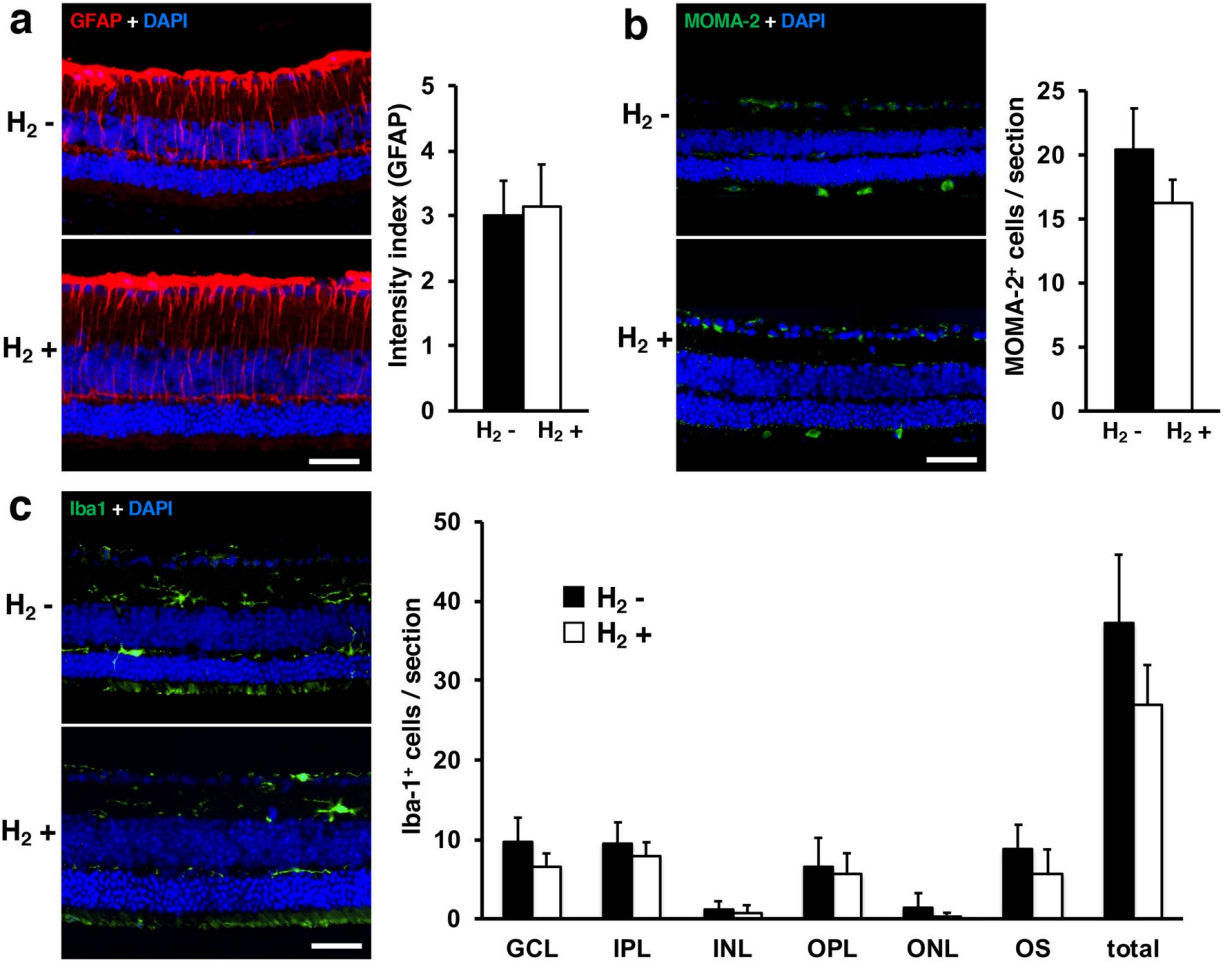
Immunohistochemical analysis of GFAP, MOMA-2, and Iba-1. Immunohistofluorescence for GFAP (**a**), MOMA-2 (**b**), and Iba-1 (**c**) are shown in representative retinal specimens with/without H_2_ water (each group: n = 4). We found no significant difference between with/without H_2_ water (GFAP: p = 0.64, MOMA-2: p = 0.06). (**c**) Mean numbers of microglial cells counted in each retinal layer and total. No significant differences between with/without H_2_ water were evident. Layers were defined as: ganglion cell layer (GCL); inner plexiform layer (IPL); inner nuclear layer (INL); outer plexiform layer (OPL); outer nuclear layer (ONL); and layer of the photoreceptor outer segments (OS). Scale bar 50 μm. Bars depict means ± standard deviation (SD).

We also carried out immunohistochemical analyses of retinal cross-sections to evaluate the effect of H_2_ water on retinal inflammatory response in rd6 mice. Figure 7 shows representative retinal cross-sections in which cells were stained for GFAP, MOMA-2, and Iba-1, with nuclei stained using DAPI (blue). Mean GFAP intensity was 3.01 ± 0.5 in the control group and 3.15 ± 0.64 in the H_2_ group (p = 0.64; Fig. 7a). The number of MOMA-2-positive cells per section was 20.38 ± 3.25 in the control group and 16.29 ± 1.76 in the H_2_ group (p = 0.06; Fig. 7b). Numbers of microglial (Iba-1-positive) cells were subsequently counted in each retinal layer: ganglion cell layer (GCL); inner plexiform layer (IPL); inner nuclear layer (INL); outer plexiform layer (OPL); outer nuclear layer (ONL); and layer of the photoreceptor outer segment (OS). Mean numbers of microglia in the control/H_2_ groups were 9.75 ± 2.98/6.5 ± 1.73 in GCL, 9.5 ± 2.65/8 ± 1.63 in IPL, 1.25 ± 0.96/0.75 ± 1.96 in INL, 6.5 ± 3.7/5.75 ± 2.5 in OPL, 1.5 ± 1.73/0.25 ± 0.5 in ONL, 8.75 ± 3.1/5.75 ± 3 in OS, and 37.25 ± 8.6/27 ± 5 in total. No layers showed a significant difference between groups (Fig. 7c).

## Discussion

This study shows that drinking H_2_ water delayed retinal degeneration in rd6 mice. H_2_ water inhibited photoreceptor death. We found that a high concentration (1.2–1.6 ppm) of H_2_ in drinking water led to neuroprotective effects. H_2_ water also increased expression of genes of phototransduction in photoreceptors. Thus, our study may pave the way toward a new neuroprotective strategy using H_2_ water in RP patients.

In rd6 mice, the photoreceptor cell outer segments are reduced slightly in length, with a decrease in the number of photoreceptor cells^31^. As shown in Fig. 2, outer retina thickness decreased in the control group, but this decrease was significantly suppressed beginning at postnatal 21 weeks in the hydrogen drinking group. Similarly, the same result was obtained with histopathology in the analysis of outer retina thickness (Fig. 4), and a significant effect of protecting photoreceptor cells was observed in terms of photoreceptor thickness, outer retina thickness, and the number of photoreceptor cells.

We found a significant difference in rod response of ERG (0.02 cd·s/m^2^; Fig. 3). Previous studies have shown that the decreased ERG amplitude in rd6 mice is detected beginning at P25^36^ to 70 weeks of age^31^, and the rod response reduces earlier than the cone response^36^. Our data showed the rod function could be rescued by H_2_ water, whereas a slight effect was observed for the mixed rod-cone function. Longer observation may be required to determine the effect of the H_2_ water for mixed rod-cone function.

We investigated two cone opsins, long wavelength-sensitive red and green opsin, and short wavelength-sensitive blue opsin. The number of red and green opsin-positive cells in the H_2_ group was significantly higher than that in the control group (Fig. 5a). The number of blue opsin-positive cells in the H_2_ group tended to be higher than that in the control group (Fig. 5b). In RP, not only rod cells but also cone cells disappear, and drinking H_2_ water was effective in suppressing the decrease in cone cells in rd6 mice.

The rd6 mouse has a mutated *MFRP* and 4-bp deletion in a splice donor sequence, resulting in exon 4 being skipped and a truncated protein^25^. Although *MFRP* mutations are linked to photoreceptor cell degeneration, MFRP protein function is not completely understood^32^. In this RNA-seq experiment, expression of the phototransduction gene cluster in the hydrogen group was increased (Fig. 6). When exposed to light, rhodopsin is activated, and phosphodiesterase is activated via transducin, thereby degrading the second messenger cyclic guanosine monophosphate. As a result, the cyclic nucleotide-gated channel in the plasma membrane closes, and inward current stops flowing, resulting in a decrease in the membrane potential. Phototransduction in photoreceptor cells is illustrated in Fig. 6c. Expression of genes related to phototransduction such as rhodopsin, transducin, phosphodiesterase, and cyclic nucleotide-gated channel was elevated. Drinking H_2_water contributed not only to neuroprotection of photoreceptor cells but also to improvement in photoreceptor function.

We examined the superpathway of cholesterol biosynthesis, which was an upregulated pathway in the H_2_ group. *Idl1*, *Acta2*, *Cyp51A1*, and *Hmgcs1* were slightly elevated (Supplementary Fig. S1). Our gene expression analysis also revealed that the pathway of estrogen receptor signaling was slightly downregulated in the H_2_ group. Although an influence of sex on phenotype was not observed in rd6 mice, the possibility of different responses to H_2_ treatment cannot be ruled out, due to differences in hormonal profiles and inflammatory responses. As our study used only male mice, further research is needed to clarify potential sex differences in H_2_ effects.

The inflammatory response and response to oxidative stress were expected to be affected, but the genes involved in these responses were not significantly changed. For the RNA-seq data of this experiment, we analyzed the whole neural retina, but if we had performed single-cell RNA-seq with photoreceptor cells, different results may have been obtained. In addition, the analysis was performed at 49 weeks of age, but because photoreceptor degeneration has progressed considerably, performing the analysis at an early stage when photoreceptor cells remain may be important.

In this experiment, as shown in Fig. 7, we found no difference in the average expression intensity of GFAP- and MOMA-2-positive cells. GFAP is an intermediate filament protein and is a marker of Müller glial cells, but its expression is increased by inflammation. However, unlike during acute inflammation, inflammation is minimal in RP, and a change in GFAP expression is unlikely. MOMA-2 is a monocyte/macrophage-specific protein. MOMA-2 positive cells are found in the subretinal space of rd6 mice. In our experiment, we found no significant difference in the number of MOMA-2 positive cells between the H_2_ group and the control group (Fig. 7b).

Microglia are involved in the progression of RP and pathologically accumulate in the outer layer of the retina in RP mice^37,38^. Mechanistically, microglia have been shown to play a key role in photoreceptor degeneration in RP^39^. Iba-1 is a microglia/macrophage-specific calcium-binding protein with actin-bundling activity, and shows increased expression during neuroinflammation. We assessed the microglial infiltration into the outer layer of the retina by counting Iba1+ cells, but found no significant change in the H_2_ group. Further evaluation of the effect of hydrogen on microglial inflammatory response in rd6 mice will require specific evaluation of inflammatory gene expression in microglia.

As an animal model with slow retinal degeneration, rd6 is suitable for observing the effects of long-term interventions^31^. Hadziahmetovic et al. reported that 5 months of treatment with the oral iron chelator deferiprone (DFP) prevented thinning of the outer retina in rd6 mice^40^. As DFP appears effective in the reversal of oxidative stress-related tissue damage, the mechanism by which DFP delays the progression of RP might be similar to that of hydrogen. The present study evaluated the therapeutic effects of antioxidant therapy on RP progression from multiple perspectives, including not only morphological changes, but also by ERG and RNA-seq.

Gene and cell therapy has also been investigated in rd6 mice, and intravitreal injection of genetically engineered bone marrow-derived mesenchymal stromal cells (MSCs) deigned to overexpress brain-derived neurotrophic factor (BDNF) resulted in rescue from the chronic degenerative process of slow retinal degeneration in recipient rd6 mice^41^. These findings suggested that anti-apoptotic signaling induced by MSC-BDNF rescued retinal cells. Our RNA-seq data showed upregulation of anti-apoptotic factor *Bcl2* in the H_2_ group (Supplementary Fig. S1), suggesting that hydrogen therapy may also be involved in the inhibition of apoptosis in rd6.

In P23H-1 and Royal College of Surgeons (RCS) rats as other animal models, administration of basic fibroblast growth factor (FGF2) and minocycline has been shown to increase photoreceptor survival. Minocycline reduced microglial activation and migration, and the combination of FGF2 and minocycline exhibited greater neuroprotective effects than the effects of either agent alone^42^. The therapeutic effects of combined administration of hydrogen and treatments with different mechanisms of action clearly merits further research.

Currently, H_2_ can be administered via multiple routes. In clinical applications, the common routes of H_2_ administration include H_2_ gas, drinking H_2_-rich water, injection of H_2_-rich saline, bathing in H_2_ water, H_2_ intake of a solid carrier (coral calcium hydride), and ocular instillation of H_2_-rich saline. Previously, we reported that ocular instillation of H_2_-rich saline is a useful therapy for retinal artery occlusion^12^. Ocular instillation of H_2_-rich saline is effective for sudden onset acute diseases such as retinal artery occlusion. However, drinking water is more effective for chronic diseases such as RP because eye drops cannot be applied all the time. In this experiment, we planned our study using drinking water. Shimouchi et al. reported that after the intake of 500 ml of H_2_ water, the concentration of H_2_ in the breath increases to the level of 36 ppm after 10 min and gradually decreases to the baseline level of 7 ppm at 60 min^43^. Sano et al. reported that 60 min after a single dose of hydrogen, the blood hydrogen concentration is higher than the steady state^44^. We consider that a small amount of drinking water can sufficiently supply hydrogen to the retina. To further improve the effect, the combined use of inhalation of hydrogen during sleep may also prove effective.

H_2_ has been reported as a novel potential therapeutic strategy for the prevention and treatment of chronic neurological diseases, including AD^45,46^, cognitive dysfunction^47^, mood disorders^48,49^, and PD^50^. We hope that H_2_ will play a similar role for RP.

## Methods

### Animals

Male C57BL/6J mice from Charles River Laboratories Japan (Tokyo, Japan) were used to examine changes in hydrogen concentration during drinking of H_2_ water (Fig. 1). Male rd6 mice from The Jackson Laboratory (Bar Harbor, ME) were used for the other experiments. From postnatal 4 weeks, rd6 mice started to drink either regular water or H_2_ water. Mice were housed individually in standardized laboratory conditions and given tap water and food ad libitum. All animals were treated in accordance with the ARVO Statement for the Use of Animals in Ophthalmic and Vision Research. The studies were approved by the Animal Care and Use Committee of Nippon Medical School (approval number; H28-049, 2021-021). All experiments were performed in accordance with the ARRIVE guidelines.

### Preservation of the H_2_ concentration in H_2_ water

Four male 11-week-old C57BL/6J mice were allowed to drink H_2_ water (1.2–1.6 ppm of hydrogen) in an aluminum pack (Merodian Co., Osaka, Japan) for 1 week. Hydrogen leaks rapidly from the H_2_ water in regular drinking bottles or valves, so to ensure that mice in this experiment drank a stable, high concentration of H_2_ water, we developed unique water drinking valves, which were designed to completely match the water outlet of the H_2_ water in an aluminum pack and prevent gas from leaking. In addition, a backflow prevention valve was incorporated into the water drinking valve to prevent air from entering the package. The H_2_ concentration was measured before drinking and 1 week after drinking using a needle-type H_2_ sensor (Unisense, Aarhus N, Denmark).

### OCT imaging

Mice were anesthetized, and pupils were dilated. Mice were placed on the rodent alignment stage. An ophthalmic viscosurgical device was applied with cover glass. OCT images were acquired using a Cirrus HD-OCT Model 4000 (Carl Zeiss, Oberkochen, Germany). A specific adaptor including a 90D lens was placed on the objective lens of the Multiline OCT to focus on the mouse retina. The OCT image resolution was 500 pixels (height) × 750 pixels (width). All images were location matched by scanning vertically through the center of the optic nerve head. The average thickness of the outer retina (between the outer plexiform layer and the RPE) was measured at 200 pixels from the optic nerve head using Adobe Photoshop (Adobe Inc., San Jose, CA). In this study, the maximum number of B-scans set by the manufacturer (20 times) was used for averaging. Experimental (n = 10) and control (n = 8) eyes from each mouse were compared at postnatal 6, 11, 21, 32, 39, and 47 weeks.

### ERGs

After overnight dark adaptation, mice were anesthetized with an intraperitoneal injection of normal saline solution containing ketamine (80 mg/kg) and xylazine (10 mg/kg). ERGs were recorded using a synchronized trigger and summing amplifier (Primus; Mayo, Nagoya, Japan) with a stimulation device (LS-W; Mayo), as described in our previous report^51,52^. After pupil dilation (0.5% tropicamide and 0.5% phenylephrine ophthalmic solution; Santen Pharmaceutical Co., Osaka, Japan), scotopic responses were examined. ERG responses were measured according to the International Society for Clinical Electrophysiology of Vision guidelines. Scotopic-adapted standard white flash stimuli were set at 0.02 cd·s/m^2^ and 2 cd·s/m^2^. At least three ERG readings were collected from each eye. Experimental (n = 10) and control (n = 8) eyes from each mouse were compared at postnatal 5, 10, 16, 20, 27, and 42 weeks.

### Histology and thickness of the outer retina

At 49 weeks of age, experimental (n = 4) and control (n = 4) eyes were enucleated and fixed overnight in 4% paraformaldehyde in 0.1 M phosphate-buffered saline (PBS) at 4 °C as described in our previous report^53,54^. Briefly, the eyes were sequentially transferred to PBS containing sucrose. After the anterior segments were removed, the eye cups were frozen. Six-micrometer cryostat sections were cut in a plane parallel to the vertical meridian of the eye. To measure retinal thickness, sections were stained with hematoxylin and eosin. Retinal thickness, defined as the total width between outer nuclear layer cells and intact outer segments, was then measured. These measurements were made in an area 1 mm from the optic disc using a light microscope, and the thicknesses measured in three different sections were averaged at a final magnification of 40× using a light microscope and image analysis software (Photoshop, Adobe Inc.).

### Immunohistochemistry

For immunohistochemistry, 6-μm-thick sections of retina in the plane of the mid-optic disc were stained. For rhodopsin and opsin double immunostaining, sections were incubated with HistoVT One (Nacalai Tesque, Kyoto, Japan) at 70 °C for 20 min. For rhodopsin staining, a mouse-on-mouse Kit (Vector Laboratories, Burlingame, CA) was used according to the manufacturer's instructions. Rhodopsin (1D4) monoclonal mouse antibody (1:1000) (Abcam, Cambridge, UK) and streptavidin-Cy3 (1:2000) (Thermo Fisher Scientific, Waltham, MA) were used. For opsin immunostaining, sections were incubated with 10% donkey serum in PBS at room temperature (RT) for 1 h. Rabbit anti-opsin red/green antibody (Merck, Kenilworth, NJ) or rabbit anti-opsin blue antibody (Merck) was applied at 4 °C overnight. Alexa fluor 488 donkey anti-rabbit IgG (1:500) was applied at RT for 2 h. Sections were then mounted using a medium containing 4,6-diamidino-2-phenylindole (Vector Laboratories) and observed under a fluorescence microscope (IX83; Olympus, Tokyo, Japan). Images of the whole retina were captured at 40× magnification and tiled automatically, and double-positive cells were counted for each retina using image analysis software (cellSens Dimension; Olympus). For GFAP and Iba-1 staining, sections were similarly incubated with HistoVT One (Nacalai Tesque), then incubated with 10% donkey serum in PBS containing 0.1% Triton X-100 at RT for 1 h. Rabbit anti-GFAP antibody (1:500) (DAKO, Santa Clara, CA) or rabbit anti-Iba-1 antibody (1:500) (Fujifilm, Tokyo, Japan) was applied at 4 °C overnight. Alexa fluor 488 donkey anti-rabbit IgG (1:500) was applied at RT for 2 h. Alexa fluor 568 donkey anti-rabbit IgG (1:500) for GFAP and 488 donkey anti-rabbit IgG (1:500) for Iba-1 were applied at RT for 2 h. GFAP expression was analyzed using ImageJ software (version 1.52; NIH, Bethesda, MD), as previously described^52,55^. Each image was captured using the same camera settings for gain and time. Data were obtained for each region of interest based on pixel intensity from each group (n = 4). Quantitation was performed in a blinded manner. For MOMA-2 staining, sections were similarly incubated with HistoVT One (Nacalai Tesque). Endogenous biotin was blocked using an Avidin/Biotin Blocking Kit (Abcam) for 10 min. Then, sections were incubated with 10% goat serum in PBS containing 0.1% Triton X-100 at RT for 1 h. Rat anti-MOMA-2 antibody (1:90) (Merck) was applied at 4 °C overnight. Sections were then incubated with goat biotinylated anti-rat IgG (1:150) (Vector Laboratories) for 1 h at RT. Streptavidin Alexa Fluor-488 conjugate (1:500) (Invitrogen) was applied at RT for 2 h. MOMA-2-positive cells between the photoreceptor and RPE were counted. Numbers of microglial (Iba-1-positive) cells were subsequently counted in each layer: GCL, IPL, INL, OPL, ONL, and OS. These counts were pooled to obtain a mean number of microglial cells per layer and per retinal section (n = 4).

### RNA-seq and DEG analysis

Total RNA was extracted from each sample (H_2_ group: n = 3; control group: n = 4) of neural retina, treated with DNase 1, and purified using a RNeasy Mini Kit according to the manufacturer’s instructions (Qiagen, Valencia, CA). Libraries were sequenced (150 bp × 2 paired-end) on a Novaseq 6000 (Illumina, Inc. San Diego, CA) with a depth of > 40 million reads. Library preparation and sequencing procedures were performed by Rhelixa (Tokyo, Japan), a company specializing in life sciences. Data quality of raw RNA-seq reads in FASTQ files was assessed using FastQC (ver. 0.11.7) to identify potential sequencing cycles with low average quality and base distribution bias. Reads were processed with Trimmomatic (version 0.38), allowing spliced read alignment to the mouse reference genome (GRCm38: mm10) using HISTAT2 (ver. 2.1.0). Fragments per kilobase of exon per million reads mapped (FPKM), FPKM-upper quartile (UQ), and transcripts per million (TPM) data were calculated using featureCounts (version 1.6.3) from the mapped reads. FPKM values were analyzed using iDEP, an integrated web application for RNA-seq data analysis^56^. DEG between H_2_-treated and control groups were identified using the two-tailed permutation FDR-based Student’s t test (FDR < 0.15). We then performed a pathway analysis based on the identified genes and generated images using QIAGEN IPA (Ingenuity^®^ Systems, www.ingenuity.com).

### Statistics

All comparisons between the control group and H_2_ group were done with the paired *t*-test. The mean and standard deviation for these measurements were calculated for each group. Values of p < 0.05 were considered statistically significant.

### Ethics approval

All animals were treated in accordance with the ARVO Statement for the Use of Animals in Ophthalmic and Vision Research. The studies were approved by the Animal Care and Use Committee of Nippon Medical School (approval number; H28-049, 2021-021).

## Supplementary Information

Supplementary Information.

## Data Availability

The datasets generated during and/or analyzed during the current study are available from the corresponding author on reasonable request.
