# Supplementary material for: Drinking hydrogen water improves photoreceptor structure and function in retinal degeneration 6 mice
Source: Sci Rep. 2022 Aug 10;12:13610. doi: 10.1038/s41598-022-17903-8 (PMC9365798; doi:10.1038/s41598-022-17903-8)
Supplement: Supplementary file 1 — Supplementary Information. [file 41598_2022_17903_MOESM1_ESM.pdf]

Supplementary Information

Drinking hydrogen water improves photoreceptor structure and function in retinal degeneration 6 mice

Tsutomu Igarashi*, Ikuroh Ohsawa, Maika Kobayashi, Kai Miyazaki, Toru Igarashi, Shuhei Kameya, Asaka Lee Shiozawa, Yasuhiro Ikeda, Yoshitaka Miyagawa, Mashito Sakai, Takashi Okada, Iwao Sakane, Hiroshi Takahashi

*Corresponding author

Address for reprints: Tsutomu Igarashi, MD, PhD

Department of Ophthalmology, Nippon Medical School Chiba Hokusoh Hospital, 1715, Kamakari, Inzai City, Chiba 270-1694, Japan

E-mail: tutomu@nms.ac.jp

Supplementary Figure S1

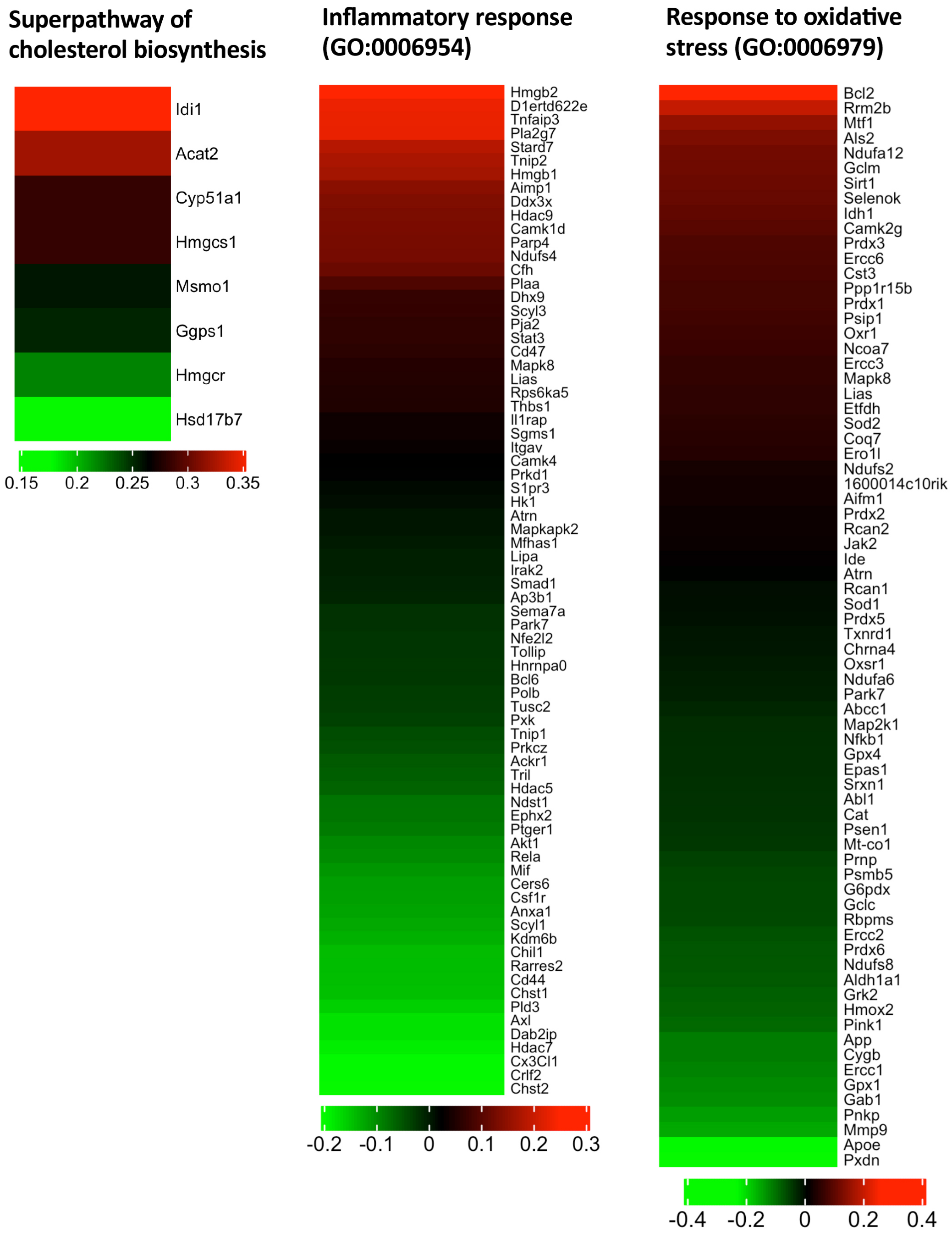

Supplementary Figure S1. Heatmap of the superpathways of cholesterol biosynthesis, inflammatory response (GO:0006954), and response to oxidative stress (GO:0006979). Red and green colors indicate genes that are upregulated or downregulated, respectively, in the H_2 group.
